# Screening and Evaluation for Antixenosis Resistance in Wheat Accessions and Varieties to Grain Aphid, *Sitobion miscanthi* (Takahashi) (Hemiptera: Aphididae)

**DOI:** 10.3390/plants11081094

**Published:** 2022-04-18

**Authors:** Kifle Gebreegziabiher Gebretsadik, Yong Zhang, Julian Chen

**Affiliations:** 1 State Key Laboratory for Biology of Plant Diseases and Insect Pests, Institute of Plant Protection, Chinese Academy of Agricultural Sciences, Beijing 100193, China; kiflegeb2004@gmail.com; 2 Tigray Agricultural Research Institute, Tigray +492, Ethiopia

**Keywords:** *Sitobion miscanthi*, *Triticum aestivum*, wheat accessions and varieties, antixenosis resistance, electronic penetration graph (EPG)

## Abstract

The grain aphid, *Sitobion miscanthi* causes serious damage by removing nutritional content from wheat plants and transmitting viral diseases. The use of resistant wheat cultivars is an effective method of aphid management. To identify *S. miscanthi* resistant cultivars, preliminary antixenosis resistance screening was conducted on 112 Ethiopian and 21 Chinese wheat accessions and varieties along with bioassay to test for further antixenosis resistance, identification of aphid feeding behavior using electrical penetration graph (EPG), and imaging of leaf trichome densities using a 3D microscope. According to antixenosis resistance screening, one highly-resistant, 25 moderately-resistant, and 38 slightly-resistant wheat cultivars to *S. miscanthi* were identified. Aphid choice tests showed that Luxuan266, 243726, and 213312 were the least preferred after 12, 24, 48, and 72 h of *S. miscanthi* release. Longer duration of Np, longer time to first probe, and shorter duration of E2 waveforms were recorded in Lunxuan266, 243726, and 213312 than in Beijing 837. The trichome density on adaxial and abaxial leaf surfaces of Lunxuan266, 243726 and 213312 was significantly higher than on those of Beijing 837. We concluded that Lunxuan266, 243726, and 213312 were antixenosis resistant to *S. miscanthi* based on the choice test, EPG results, and leaf trichome densities.

## 1. Introduction

Wheat (*Triticum aestivum)* is a major and healthy cereal crop that is consumed as a staple human food in many parts of the world [1]. China is among the major producers and consumers of wheat in the world [2]. Wheat is cultivated in almost all provinces of China and its production has rapidly grown within the past thirty years [3]. Ethiopia is one of the largest wheat producers in sub-Saharan countries [4]. Wheat accounts for about 18% of total cereal production and is the main crop for food security in Ethiopia [5]. However, the production and productivity of wheat are highly challenged by biotic and abiotic stresses. Insect pests and diseases are among the biotic stresses that cause wheat damage [6]. Among insect pests, aphids cause an average yield loss of 15% in China [7] Aphids are a major agricultural insect pest throughout the world. Cereal aphid causes serious damage by removing nutritional contents from the host plant [8]. They are also vectors of the barley yellow dwarf virus [9]. Aphids penetrate the plant epidermis with their stylet and push it through the parenchyma extracellular tissue until they reach the vascular bundles. When probing and feeding, aphids secrete saliva containing effector enzymes into their host plants to change the host cell’s processes and facilitate infestation [10]. Gel and watery saliva are the key factors in the process of aphid–plant interactions [11]. Watery salivary is used to detoxify secondary metabolites such as plant phenols and alkaloids [11]. The aphid clones of some species differ in probing and feeding behavior on some infested plants [12]. Aphid feeding increases the activity of superoxide dismutase, glutathione reductase, phenylalanine ammonia-lyase, and polyphenol oxidase enzymes of the wheat plant to defend against aphid damage [13]. A higher number of pre-alighting aphid deterring signals are present in older wheat plants compared to younger plants [14].

To defend against insects and insects, plants develop counter-defenses in the plant–insect interaction [15]. Wheat cultivars’ variation in resistance can arise from aphids’ ability to attack the plant and from the plant’s ability to defend from aphid attack [8]. The defense responses of host plants to aphids occur through antixenosis, antibiosis, tolerance, and a combination of the three mechanisms [16,17]. Antixenosis is a plant characteristic that negatively affects the host-finding and acceptance processes of insects [18]. Plants develop defensive chemicals such as secondary metabolites, protease inhibitors, plant volatiles, surface strictures, and extra-floral nectar to prevent insect pests [19]. Antixenosis resistance is mainly associated with the production of volatile organic compounds (VOCs) such as (*E*)-b-farnesene, (Z)-3-hexenal, and (*E*)-2-hexenal [20]. These VOCs released by host plants can repel herbivores or attract natural enemies of herbivores [21]. The position and density of trichomes function as physical barriers to prevent aphids from feeding on the adaxial leaf surfaces of the wheat plant [22].

Plant volatile compounds have been found to be stored in plant tissues and/or induced after herbivore damage [23]. Host plant resistance to insects, particularly induced resistance, can also be manipulated with the use of chemical elicitors of secondary metabolites, which confer resistance to insects [21]. The application of biotic and abiotic stresses on wheat plants has an additive effect on volatile organic compound emissions [24]. Various abiotic factors such as temperature, light intensity, water, and nutrient availability affect the constitutive emission of plant volatiles [23]. Most volatiles such as monoterpenes, diterpenes, and sesquiterpenes are released after herbivore damage [25]. Hordenine and gramine alkaloids are the best herbivore-feeding deterrents in cereal crops [26]. Increasing crop diversity in the same field reduces pest populations and increases natural enemies [27]. The intercropping of wheat plants with volatile-releasing plants has repellent and toxic effects, masks host plant odors, affects aphid’s visual orientation, stimulates natural enemies, and induces resistance [27]. The application of garlic oil blend and diallyl disulfide to wheat fields reduces aphid populations and increases their natural enemies [28].

The grain aphid, *S. miscanthi,* is among the cereal aphid species found distributed worldwide, especially in temperate climates [29]. In China, *S. miscanthi* is classified into five biotypes based on responses to wheat cultivars [30]. This species is a major pest of wheat in the northern and central parts of China [29]. Geographic variations, photoperiod, and temperature affect the life cycle patterns of *S. miscanthi* [31]. The use of a resistant wheat cultivar is an effective method of cereal aphid management. Many types of research have been done to identify aphid-resistant wheat varieties in China. Some wheat varieties with resistance to *S. miscanthi*, such as Amigo, Fengchan NO.3, Zhongsiwumang, JPI, KOK, Jinmai 3I, and L1, have been identified [30]. However, there is a lack of research findings related to *S. miscanthi*-resistant wheat verities. This study aims to identify new *S. miscanthi*-resistant wheat cultivars from Ethiopian and Chinese wheat accessions and varieties, and then to develop a potential tactic of resistant wheat application to achieve aphid control and reduce pesticide use and yield loss in the future. The specific objectives are as follows. The first one is to identify antixenosis resistant wheat cultivars to *S. miscanthi* from 112 Ethiopian and 21 Chinese wheat accessions and varieties. The second objective is to determine the relative attractiveness of *S. miscanthi* among highly and moderately resistant wheat accessions and varieties. The third is to record the feeding behavior of *S. miscanthi* on the wheat accessions and varieties with antixenosis resistance using the electronic penetration graph (EPG) technique. The last objective is to determine leaf trichome densities of the wheat accessions and varieties with antixenosis resistance at the seedling stage.

## 2. Results

### 2.1. Preliminary Antixenosis Resistance Screening

The results of preliminary antixenosis resistance screening after 24 and 48 h of winged S. miscanthi release are presented in Table 1. After 48 h from winged aphid release, the 136 wheat accessions and varieties were categorized into one highly-resistant variety (Lunxuan 266), 25 moderately-resistant accessions and varieties (five Chinese and 20 Ethiopian), and 38 slightly-resistant accessions and varieties (eight Chinese and 30 Ethiopian), 29 slightly-susceptible accessions and varieties (four Chinese and 25 Ethiopian), 19 moderately-susceptible accessions and varieties (1 Chinese and 18 Ethiopian), and 21 highly-susceptible accessions and varieties (3 Chinese and 18 Ethiopian). Then, differences in the number of winged *S. miscanthi* settling on the wheat accessions and varieties were evaluated. The numbers of winged *S. miscanthi* settling on the wheat accessions were clustered using the heatmap TBtool for additional clarification of the antixenosis resistance screening (Figure S1). Groups 1 and 2 were comprised of 10 and 23 wheat accessions, respectively, with high and moderate antixenosis resistance, such as Lunxuan 266, 243726, and 213312. Significant differences in the mean number of *S. miscanthi* populations were observed among the screened wheat accessions and varieties.

Table 2 shows the mean number of adult *S. miscanthi* settling per plant within 48 h and produced per plant within 28 days in the antixenosis and antibiosis resistance screening of wheat accessions and varieties. The results indicated that the wheat accessions and varieties were divided into high and moderate antixenosis resistance groups, whereas in the case of antibiosis resistance, these wheat accessions and varieties were grouped into slightly resistant, slightly susceptible, and moderately susceptible. Therefore, the antixenosis and antibiosis resistance mechanisms of these wheat accessions and varieties were not found to be on the same level of the resistance scale.

### 2.2. Choice Test for Further Antixenosis Identification

**Figure 1:**
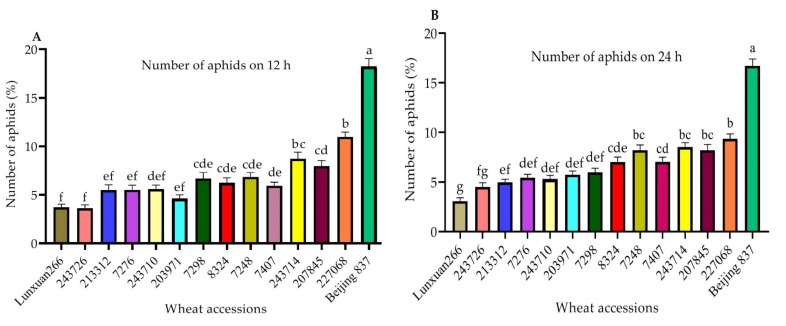

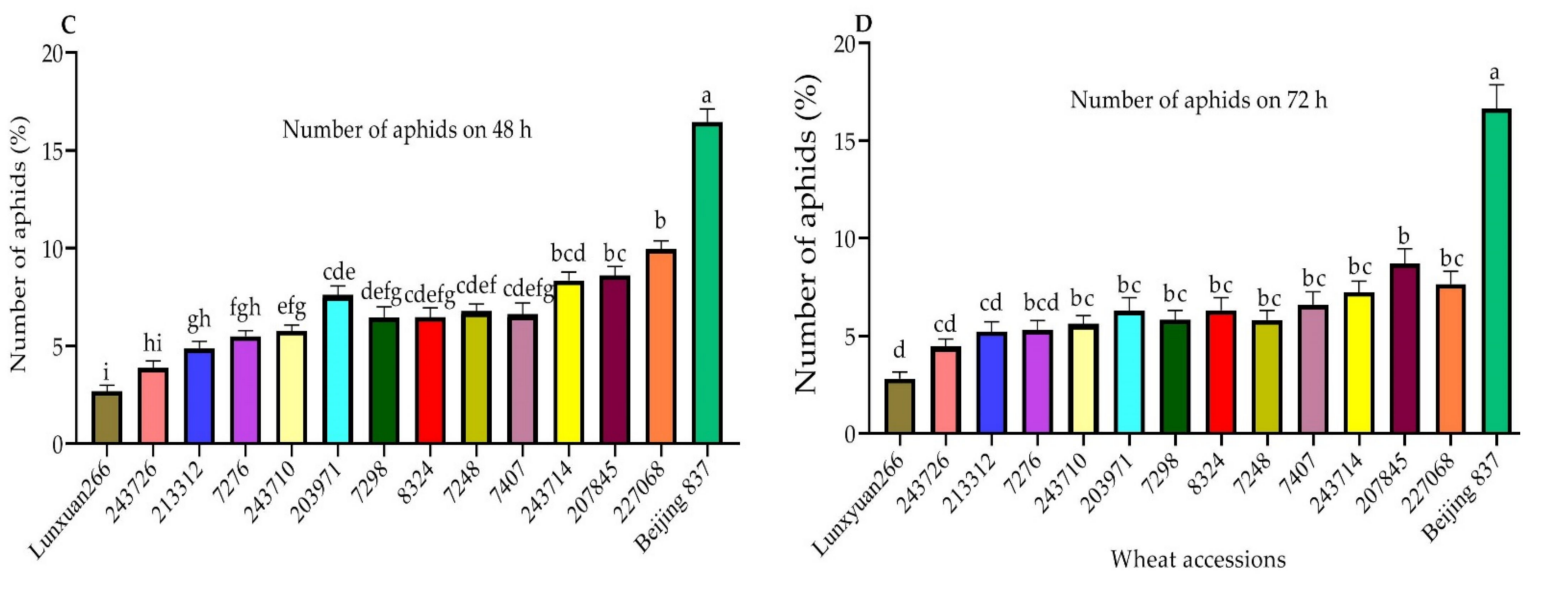
Percentage of winged *S. miscanthi* settled on the wheat accessions 12, 24, 48, and 72 h after being released. (**A**–**D**) indicate the percentage of winged *S. miscanthi* settled on wheat plants after 12, 24, 48 and 72 h, respectively. Bars represent the mean percentage of aphids settled (mean ± ME, *n* = 16). Different letters above the bars indicate significant differences.

There was a significant difference (*p* < 0.05) in the percentage of winged *S. miscanthi* settling on the 14 wheat accessions and varieties tested 12, 24, 48, and 72 h after aphid release (Figure 1A–D). All of the tested accessions and varieties showed significant differences from Beijing 837. After 12 h., the percentage of aphids settled on Lunxuan266, 243726, 213312, and 7276 was significantly lower than the percentage settled on 243714, 207845, 227068, and Beijing 837 (Figure 1A). After 24 h, the percentage of aphids settled on Lunxuan266, 243726, and 213312 was significantly lower than the percentage settled on 7248, 7407, 243714, 207845, 227068, and Beijing 837 (Figure 1B). After 48 h, the percentage of aphids settled on Lunxuan266, 243726, and 213312 was significantly lower than the percentage settled on 203971, 243714, 207845, 227068, and Beijing 837 (Figure 1C). After 72 h, the percentage of aphids settled on Lunxuan266, 243726, and 213312 was significantly lower than the percentage settled on 207045 and Beijing 837 (Figure 1D). The results of the choice test indicated that Lunxuan266, 243726, and 213312 were the least preferred plants among the 14 wheat accessions and varieties tested.

### 2.3. Measuring Sitobion miscanthi Feeding Behavior by EPG

As shown in Table 3, there were significant differences among the wheat accessions and varieties for the EPG parameters associated with the total duration of Np, time to the first probe, duration to first probe, mean duration of E1, time to the first E2, number of E2, the total and mean duration of E2, and time to first sustained E2 (≥10 min) (*p* < 0.05). The total duration of the Np for Lunxuan266, 243726, and 213312 was longer than that of Beijing 837. The time to the first probe of *S. miscanthi* feeding on Lunxuan266 and 243726 was longer than that of Beijing 837. The mean duration of E1 waveforms of *S. miscanthi* feeding on 213312 and Lunxuan266 was significantly longer than that of Beijing 837. The time to reach the first E2 and first sustained E2 (≥10 min) was longer on Lunxuan266 than on Beijing 837. Furthermore, the total and mean duration of E2 waveforms of *S. miscanthi* feeding on the test accessions was shorter than on Beijing 837. Therefore, the test accessions showed epidermal and phloem-based resistance to *S. miscanthi* damage.

### 2.4. Trichome Density

**Figure 2:**
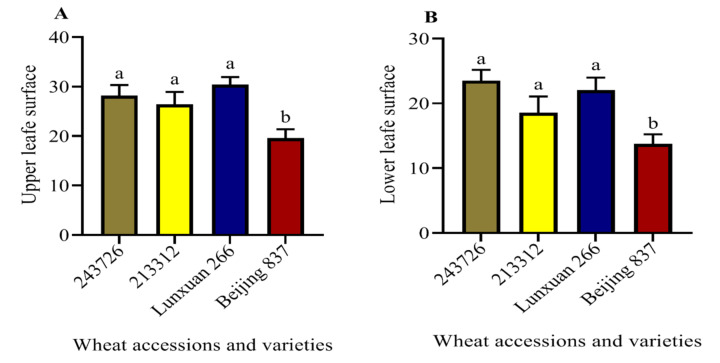
Trichome density of four wheat leaves. Bars represent the average number of trichome densities per mm^2^ (mean ± SE, *n* = 10). **A** and **B** indicate trichome density on the upper (adaxial) and lower (abaxial) leaf surfaces, respectively. Different letters above the bars indicate significant differences.

As shown in Figure 2, the trichome density on the upper (adaxial) leaf surfaces of wheat accessions and varieties with antixenosis resistance (Lunxuan266, 243726, and 213312) was significantly higher than on Beijing 837 (*p* < 0.05). Furthermore, significantly higher trichome densities were recorded on the lower (abaxial) leaf surface of Lunxuan266, 243726, and 213312 than on Beijing 837 (*p* < 0.05). The variations in trichome density could be caused by the genetic differences among the evaluated wheat plants. As a result, the increased trichome densities on the test accessions and varieties act as a physical barrier, preventing aphid-stylet penetration for feeding.

### 2.5. Correlation Analysis between Number of Winged Sitobion micanthi Settling and EPG Parameters on Wheat Seedlings

As shown in Table 4, the number of winged *S. miscanthi* settling on wheat leaves 12, 24, 48, and 72 h after release was negatively correlated with Np, E1, C, and Pd, and positively correlated with E2 and G waveforms. The number of *S. miscanthi* settling on the wheat leaves was negatively correlated with Np and E1 waveforms to a significant extent (*p* < 0.05), and positively correlated with E2 to a significant extent. These results suggest the presence of some resistance factors in the epidermis and/or mesophyll cell and phloem of wheat leaves that have repellent or deterrent effects on winged *S. miscanthi*.

### 2.6. Correlation Analysis between Number of Aphids Settling and Trichome Density on Wheat Accessions and Varieties

As shown in Table 5, the number of winged *S. miscanthi* settling on wheat leaves after 12, 24, 48, and 72 h was significantly negatively correlated (*p* < 0.05) with trichome density on the adaxial and abaxial leaf surfaces of the four wheat accessions and varieties evaluated. These results suggest that the higher trichome density on leaves of Luxyaun266, 243726, and 213312 hinders the settlement and feeding of winged *S. miscanthi*.

## 3. Discussion

### 3.1. Preliminary Antixenosis Resistance Screening

Our study suggested that some of the wheat accessions and varieties included in the antixenosis resistance screening were less preferred by the winged *S. miscanthi*, which is similar to previous findings [32]. Thus, the screened 136 wheat accessions and varieties were categorized as one highly resistant, 25 moderately resistant, 38 slightly resistant, 29 slightly susceptible, 19 moderately susceptible, and 21 highly susceptible. The smaller number of winged *S. miscanthi* settling on highly and moderately resistant accessions and varieties indicates antixenosis resistance. Many studies have demonstrated that plant olfactory cues and some chemicals such as hydroxamic acid affect the preference of aphids for plants [33]. The physical structures of plants, chemicals such as repellents and deterrents, and antifeedant effects on herbivores contribute to antixenosis resistance [34]. Most insects have innate behaviors to orient towards or away from plants using odor clues emitted by the plants; for example, European corn borer (ECB) larvae and adults accepted maize, but rejected spinach because it produces phytoecdydtrroids, which is toxic and deterrent to ECB [35]. Thus, these wheat accessions and varieties that were less preferred by *S. miscanthi* could have inherited repellent behavior.

One highly-resistant and 12 moderately-resistant wheat accessions and varieties in the antixenosis resistance screening were also evaluated for antibiosis resistance to *S. miscanthi*, and were slightly resistant, slightly susceptible, and moderately susceptible (Table 2). Our findings indicate that antixenosis and antibiosis resistance factors are not necessarily found in the same accession and that they do not depend on each other, which is consistent with previous reports [36,37].

### 3.2. Aphid Bioassays for Further Antixenosis Resistance Tests

Variations in antixenosis resistance were found among different plants of some species [38]. Plants emitted a variety of VOCs to protect themselves from herbivores, which also stimulate the neighboring healthy plants to produce defense VOCs (18). However, the amount released of each VOC varies qualitatively and quantitatively depending on the duration after herbivore damage [18,39]. The insect response to individual VOCs depends on the concentration with which the insects can be attracted or repelled [35,40]. For example, *Sitophilus granaries* and *Tribolium confusum* were repelled by blending plant VOCs (aliphatic alcohols, aliphatic aldehydes, aliphatic ketones, and aromatics) at >100 ng min^−1^ concentrations, while they were attracted at 10 ng min^−1^ concentrations [39,40]. Green leaf volatiles (GLVs) are the herbivore-induced plant volatiles (HIPVs) that attract insect herbivores directly and/or indirectly at higher concentrations [39,41]. For example, (Z)-3-hexenyl acetate, (Z)-3-hexenal, (*E*)-2-hexenal, (*E*)-2-hexen-1-ol, and (Z)-3-hexenol were attractive to ECB at higher concentrations, but repellent at low concentrations [42]. Previous studies have shown variations in antixenosis resistance among wheat cultivars for winged *S. miscanthi* settling 24 h after release [41], and 24, 48, and 72 h after release [43]. Similarly, variations were observed in some winged *Rhopalosiphum padi* and *Schizaphis graminum* settling among different wheat accessions [44,45]. Our results indicated that Lunxuan266, 243726, and 213312 were less preferred, whereas Beijing 837 was more preferred 12, 24, 48, and 72 h after winged *S. miscanthi* release. Plants with antixenosis resistance release increased VOCs in response to aphid infestation, which disturbs aphid settlement [46]. For example, VOCs such as methyl-salicylate (MeSA), linalool, and *E*-β-farnesene emitted from Prunus apices have repellent or dispersive effects on aphids [47]. The increased release of (*E*)-β-farnesene from wheat plants repels aphid pests and attracts the natural enemies of aphids [43]. Plants release EBF, which as an alarm pheromone for aphids and defends plants from aphid infestation by deterring aphids from settling and inducing more winged offspring that help to leave the plant [48]. Therefore, the variations in preference levels of winged *S. miscanthi* to the wheat accessions and varieties could be due to the variations in the types and concentrations of VOCs produced among the wheat plants evaluated.

### 3.3. Electrical Penetration Graph

Wheat plants emit repellent volatile compounds that impede aphids’ settling and feeding behaviors [49]. EPG helps to determine the effects of plant repellents and antifeedants on the feeding behavior of sap-sucking insects [50]. In our study, the total duration of Np waveforms on the test accessions was significantly longer than om Beijing 837. Similar results have been reported on Solstice, W064, W068, and W591 wheat landraces against *R. padi* [51]. The choice test for *S. miscanthi* also supported these results and indicated a clear lack of preference for the test accessions compared to Beijing 837. These results indicate the presence of repellent volatile chemicals originating from the host plant [52]. Additionally, the aphids probed more slowly on the aphid-resistance wheat accessions and varieties compared to the susceptible accession Beijing 837, which suggests that resistance factors may also be present on the surfaces of leaf tissue. This result is also supported by the higher trichome density on the leaf surfaces of aphid-resistance wheat accessions and varieties compared to Beijing 837.

The aphid *S. miscanthi* feeding on 213312 and Lunxuan 266 showed a longer total duration of E1 than on Beijing 837. The longer duration in E1 suggests the presence of some toxic and/or deterrent chemicals preventing the phloem sap from being ingested [53]. The time taken to reach the first E2 and first sustained E2 (≥10 min) on Lunxuan 266 was longer than on Beijing 837. Moreover, all of the test accessions and varieties showed a shorter total and mean duration of E2 than Beijing 837, which is coherent with the studies on host-plant resistance occurring in the phloem [53]. The shorter duration of E2 is an indication of antixenosis because the phloem activates a deterrent to aphid feeding [54]. Therefore, the longer duration of Np, time to the first probe, E1, time for the first sustained E2 (≥10 min), and shorter duration of E2 in the tested accessions indicate the antixenosis resistance to *S. miscanthi* feeding, which is most likely attributable to the presence of repellent factors in the wheat seedlings. Aphids feeding on host plants with epidermal cell and phloem-based resistance demonstrate the repellent and deterrent effects of plant repellence volatiles, which is consistent with our findings.

Some wheat volatiles are repellent to winged aphids alighting and feeding [55]. The host plants containing higher concentrations of deterrent chemicals negatively affected aphid feeding behaviors [49]. The number of winged *S. miscanthi* settling on wheat leaves 12, 24, 48, and 72 h after release showed a significant negative correlation with Np and E1, and a significant positive correlation with E2 waveforms. The results indicated that the number of winged aphids settling on the wheat accessions may be determined by repellent volatiles such as (*E*)-β-farnesene and the MeSA content of the plant, which is consistent with the previous report [56]. We have tried to collect volatile chemical components from wheat seedlings, but failed, as few volatiles were identified using GC/MS. It was speculated that the number of wheat seedlings used for emission collection was not large enough. Therefore, it is necessary to identify the volatile components of wheat that have repellent effects on *S. miscanthi* through further study.

### 3.4. Trichome Density

Trichomes are among the physical defensive mechanisms of plants, impeding insect movement, feeding, development, and reproduction [57]. The effects of such defensive structures against aphids has been reported in wheat and *E. tef* [57,58]. A higher trichome density on the upper leaf surface of the PI 137739 wheat genotype is antixenotically resistant to RWA [59]. The trichome densities of the same plant species can differ due to growth stage, genetic makeup, and leaf location, and density is also induced by insect damage [60]. In our study, we found higher trichome densities on the upper and lower leaf surfaces of Lunxuan 266, 243726, and 213312 compared to Beijing 837, which indicates that genetic variation affects trichome density. Wheat plants with dense trichomes can hamper cabbage whitefly, *Aleyrodes proletella*, from reaching its exact probing site [36]. Trichome density affects the feeding behaviors of *R. padi* and limits its performance in wheat plants [57]. Similarly, wheat cultivars with a higher number of trichomes extended the time for *R. padi* penetration [61]. In our EPG result, the time to the first probe on test accessions was longer than on Beijing 837. Thus, the cause for the lower number of winged *S. miscanthi* settling on test accessions and varieties could be caused by the higher number of trichomes on wheat seedlings. The number of winged *S. miscanthi* settled on the wheat accessions and varieties was negatively correlated with trichome density. The leaf trichome density of nine cotton varieties showed a negative correlation with aphid abundance in the plants [62]. Therefore, our results indicate that a higher trichome density on leaves may play an important role in the reduction in winged aphids settling on the tested wheat accessions and varieties.

## 4. Materials and Methods

### 4.1. Insects and Plants

The experiment was conducted inside a greenhouse at the Langfang Research Station (39°30′ N, 116°36′ E), Hebei Province, China. The grain aphids, *S. miscanthi*, used for the experiments were obtained from the laboratory of the Institute of Plant Protection, Chinese Academy of Agricultural Science (CAAS), Beijing, China. The aphid culture was kept under a greenhouse condition of temperature in the range of 20 ± 2 °C and 55–60% RH, with a photoperiod of 16:8 (L:D). The susceptible wheat variety Beijing 837 was used for the mass rearing of *S. miscanthi* [63]. Around 15 seeds of Beijing 837 were sown in a plastic pot of 10 cm diameter and kept within an aphid rearing cage sized 350 × 350 cm. Four aphid rearing cages, each containing four pots, were used for the mass rearing of *S. miscanthi*. The mass-reared winged *S. miscanthi* (emerging within 24 h) were used for the experiments. 

Wheat accessions (112 from the Ethiopian Institute of Biodiversity /EIB/, Addis Ababa, Ethiopia) and wheat varieties (21 from the Institute of Crop Sciences, CAAS, Beijing, China) were used for screening of antixenosis resistance to winged *S. miscanthi*. One highly-resistant variety (Lunxuan266) and eleven moderately-resistant accessions (243726, 213312, 7276, 243710, 8324, 7407, 203971, 243714, 7298, 207845, and 227068) from the preliminary screening were used for aphid bioassay to conduct further tests on antixenosis resistance against winged *S. miscanthi*. Both EPG recording and trichome density scanning were performed on the slightly-preferred Lunxuan266, 243726, and 213312 wheat accessions, and varieties were taken from the aphid bioassay test. Beijing 837 was included in all activities as a negative control.

### 4.2. Preliminary Antixenosis Resistance Screening of Wheat Accession and Varieties against Sitobion miscanthi

Six seeds of each of the 133 wheat accessions and varieties used for the antixenosis resistance screening were grown in plastic pots as described above, and replicated two times. After germination, the seedlings were thinned to maintain four seedlings per pot. For simplification, the 23 pots containing wheat seedlings at two-leaf visible stages (12 days after germination) [64] were grouped as one and arranged at random in a circle inside a 2 × 2 × 1.5 m rectangular gauze cage to protect against the winged aphids escaping [41]. Two hundred thirty (230) winged *S. miscanthi* having emerged within 24 h were released into the center of the experimental plants in the cage. The greenhouse was dark to avoid the artificial orientation of aphids to the screened wheat accessions and varieties in response to light. The number of adult aphids that settled on each accession and variety was counted and recorded after 24 and 48 h. The number of aphids were counted between 10:30 a.m. and 1:30 p.m., when plants are active in odor releasing.

Aphid indexes were calculated as the average number of winged aphids settled per plant of each wheat accession and variety over the average number of winged aphids settled per plant for all the wheat accessions and varieties screened. The calculated aphid index was expressed as 0 (immunity) to 6 (highly susceptible), to indicate the aphid infestation severity according to the Painter’s method [65], with the aphid index being 0 = immunity; 1 = high resistance (HR), with aphid indexes ranging from 0.01 to 0.30; 2 = moderate resistance (MR), with aphid indexes ranging from 0.31 to 0.60; 3 = slight resistance (LR), with aphid indexes ranging from 0.61 to 0.90; 4 = slight susceptibility (LS), with aphid indexes ranging from 0.91 to 1.20; 5 = moderate susceptibility (MS), with aphid indexes ranging from 1.21 to 1.50; 6 = high susceptibility (HS), with aphid indexes exceeding 1.50.

### 4.3. Aphid Bioassay for Further Antixenosis Resistance Test (Choice Test)

One highly and 12 moderately-resistant wheat accessions and varieties selected from preliminary screening, plus one susceptible variety (Beijing 837) were used for the choice test. Six seeds of each treatment were planted in each plastic pot. After germination, the seedlings were thinned to maintain four seedlings per pot. Fourteen pots containing four wheat seedlings at the two-leaf stage were randomly arranged in a circle within a rectangular gauze cage 2 m × 2 m × 1.5 m. Then, 140-winged *S. miscanthi* have emerged within 24 h were released into the center of the test plants in the cage. The greenhouse was dark to avoid the artificial orientation of aphids to the test plants in response to light. The number of aphids that settled on each treatment was counted and recorded after 12, 24, 48, and 72 h [66]. The counting was performed between 10:30 a.m. and 1:30 p.m. Each treatment was replicated 16 times. The number of winged aphids settled per treatment was divided by the total number of aphids settled per plants of each replication to calculate the proportion of *S. miscanthi* settling on each treatment.

### 4.4. Feeding Trial with EPG Recording

The feeding behavior of *S. miscanthi* on wheat leaves was recorded using the EPG (Giga-8d) method [67]. The EPG method differentiates between the feeding behaviors that indicate the acceptance or rejection of the sucking insects of the plant species, depending on variations in the duration of EPG waveforms [68]. Five seeds of each wheat accession and variety (Lunxuan 266, 243726, and 213312, and Beijing 837) were planted in a plastic pot 10 cm in diameter. Winged *S. miscanthi* were collected from the rearing cage and starved for about 1.5 h, then connected to 15 µm pieces of 2–3 cm long gold wire using water-soluble conductive silver glue. The silver glue was also used to connect the gold wire to about 3 cm copper wire, which was then connected to a brass pin. The device was attached to an eight-channel ‘Giga-8′ DC amplifier with 1 GΩ input resistance (EPG-systems, Wageningen, Netherlands) and covered within a grounded Faraday cage to protect against external noise at 21 ± 1 °C. Each aphid was placed on the first leaf of an individual eleven-day-old wheat seedling. A ground electrode was inserted into the soil of each potted plant and connected to the amplifier. Recording was performed continuously for 6 h from 9:00 to 15:00 on a daily basis. Each aphid and plant were used only once. One replicate of each of the four treatments was run per day and the positions of the plants and probe wires were randomized daily. At least 14 replicates were tested per treatment. The recorded EPG waveforms were manually analyzed using the Stylet+ software [69]. EPG parameters were selected from the Sarria Excel Notebook [70]. The waveform patterns were categorized into non-penetration (Np), stylets pathway phase (C), stylets cell punctures (Pd), xylem ingestion (G), phloem salivation (E1), and phloem ingestion (E2).

### 4.5. Trichomes Density

The trichome density of Lunxuan 266, 243726, and 213312, and Beijing 837 wheat seedlings was counted as described in [57]. Samples were collected from the second leaves of 11-day old seedlings. For trichome visualization, the middle part of the leaf sample was placed on glass microscope slides and covered with glass coverslips. The super depth-of-field 3D microscope system (vhx-20000) was used for scanning leaf trichomes. The number of trichomes was counted manually from adaxial and abaxial leaf surfaces and the density of trichomes was calculated per mm^2^. For each wheat accession and variety, five biological replicates with two images per leaf were taken.

### 4.6. Data Analysis

The number of aphids counted in the antixenosis resistance screening of wheat accessions and varieties was analyzed using heatmap clustering by TBtools [71]. All statistical analyses were conducted using SPSS 22.0. software (IBM SPSS statistics 22). Before analysis, the normality and homogeneity of variance of the data were checked. The data that did not follow normal distribution were square root transformed. Data on duration and counts were analyzed through a one-way analysis of variance (ANOVA). P values less than 0.05 were treated as statistically significant. The means were compared using Fisher’s least significance difference method for the EPG, trichome density, and aphid bioassay response variables. GraphPad Prism 8.0 was used to draw the curves and graphs for all demographic parameters.

## 5. Conclusions

We concluded that the preliminary antixenosis resistance screening showed variations in some winged *S. miscanthi* settling among the 133 wheat accessions and varieties. Out of 14 wheat accessions and varieties tested in the study, Lunxuan266, 243726, and 213312 showed the best antixenosis resistance to *S. miscanthi*. The EPG result showed a longer duration of Np and time to first probing, and a shorter duration of E2, indicating antixenosis resistance to *S. miscanthi* feeding, which is probably due to the presence of repellent factors produced from the tested wheat seedlings. The greater number of trichomes on the upper and lower surfaces of the tested wheat accessions and varieties could be among the causes for the lower number of winged *S. miscanthi* settling on the tested accessions and varieties. The overall results indicate the presence of some *S. miscanthi* repellent VOCs, as well as trichomes as physical barriers in the tested wheat accessions and varieties. It is suggested to evaluate these wheat accessions and varieties under field conditions for further confirmation of their antixenosis resistance to *S. miscanthi* infestation.

## Figures and Tables

**Table 1:** Preliminary antixenosis resistance index for *S. miscanthi* on wheat accessions and varieties.

No.	Wheat Accessions and Varieties		Resistance Scale	Aphid Index	Rank
	After 24 h	After 48 h			
1	0	0	0	0.00	Immunity
2	3	1	1	0.01–0.30	Highly resistance
3	33	25	2	0.31–0.60	Moderately resistance
4	24	38	3	0.61–0.90	Lowly resistance
5	32	29	4	0.91–1.20	Lowly susceptible
6	27	19	5	1.21–1.50	Moderately susceptible
7	14	21	6	>1.50	Highly susceptible

**Table 2:** Antixenosis and antibiosis resistance index to *S. miscanthi* for thirteen wheat accessions and varieties.

Wheat Accessions and Varieties	Antixenosis Resistance		Antibiosis Resistance	
	Resistance Index	Resistance Description	Resistance Index	Resistance Description
Lunxuan266	0.29	HR	0.67	LR
243726	0.36	MR	0.71	LR
213312	0.36	MR	1.13	LS
7276	0.43	MR	1.37	MS
243710	0.36	MR	0.67	LR
203971	0.43	MR	0.67	LR
7298	0.50	MR	1.46	MS
8324	0.36	MR	1.53	HS
7248	0.50	MR	1.08	LS
7407	0.50	MR	1.40	MS
243714	0.50	MR	0.64	LS
207845	0.43	MR	1.10	LS
227068	0.43	MR	0.73	LR

HR = Highly Resistant, MR = Moderately Resistant, LR = Less Resistant, LS = Less Susceptible, MS = Moderately Susceptible, HS = Highly Susceptible.

**Table 3:** EPG Parameters of *S. miscanthi* on seedlings of four different wheat accessions.

Aphid Feeding Parameters	Wheat Accessions or Varieties Used for the EPG Recording				*p*-Value
	213312 (MR), *n* = 20	Lunxuan266 (HR), *n* = 22	243726 (MR), *n* = 18	Beijing 837 (HS), *n* = 14	
Number of Np	3.55 ± 0.56	4.25 ± 0.44	4.62 ± 0.86	3.14 ± 0.76	ns
Total duration of Np (min)	76.99 ± 15.55 ^a^	119.06 ± 18.54 ^a^	75.48 ± 14.46 ^a^	54.8 ± 19.88 ^b^	0.0033
Mean duration of Np (min)	27.25 ± 7.01	31.47 ± 5.24	24.06 ± 6.74	30.32 ± 17.45	ns
Total number of probes	173.18 ± 20.45	148.10 ± 17.05	127.69 ± 22.56	150.50 ± 25.23	ns
Time to the first probe (min)	10.59 ± 3.75 ^ab^	25.18 ± 7.24 ^a^	23.77 ± 7.32 ^a^	6.85 ± 2.24 ^b^	0.0449
Total probing time (h)	4.59 ± 0.25	4.13 ± 0.32	4.74 ± 0.24	5.04 ± 0.32	ns
Duration of first probe (min)	2.62 ± 0.49 ^ab^	7.84 ± 4.02 ^a^	3.35 ± 1.21 ^ab^	1.58 ± 0.35 ^b^	0.0440
Number of C	85.64 ± 1.77	74.55 ± 1.81	63.92 ± 1.70	72.79 ± 1.79	ns
Total duration of C (h)	2.01 ± 0.21	1.91 ± 0.18	1.91 ± 0.19	1.47 ± 0.22	ns
Mean duration of C (min)	1.50 ± 0.19	2.38 ± 0.59	1.99 ± 0.42	1.40 ± 0.20	ns
Number of Pd	77.93 ± 9.52	67.65 ± 7.95	54.69 ± 9.81	67.93 ± 11.97	ns
Total duration of Pd (min)	7.39 ± 0.87	13.04 ± 5.52	4.71 ± 0.87	7.08 ± 1.38	ns
Number of G	2.27 ± 0.40	1.45 ± 0.21	2.00 ± 0.27	2.21 ± 0.59	ns
Total number of G (min)	57.79 ± 12.21	50.54 ± 11.02	99.01 ± 18.41	37.00 ± 8.95	ns
Mean number of G (min)	23.46 ± 4.25	34.04 ± 9.13	57.85 ± 15.90	20.16 ± 6.58	ns
Time to the first E1 (h)	2.23 ± 0.34	2.91 ± 0.43	3.05 ± 0.60	1.73 ± 0.25	ns
Number of E1	5.14 ± 0.73	3.50 ± 0.61	5.08 ± 1.48	4.14 ± 0.71	ns
Total duration of E1(min)	46.16 ± 7.01	43.01 ± 7.12	38.51 ± 8.13	29.79 ± 9.49	ns
Mean duration of E1 (min)	13.89 ± 3.41 ^a^	9.46 ± 1.91 ^a^	8.98 ± 1.79 ^ab^	3.87 ± 0.83 ^b^	0.0360
Time to the first E2	3.35 ± 0.43 ^ab^	4.64 ± 0.45 ^a^	3.61 ± 0.59 ^ab^	2.36 ± 0.34 ^b^	0.0409
Number of E2	2.22 ± 0.49 ^ab^	0.95 ± 0.31 ^b^	2.00 ± 0.65 ^ab^	3.43 ± 0.73 ^a^	0.0063
Total duration of E2 (min)	49.92 ± 12.55 ^b^	31.22 ± 13.35 ^b^	59.68 ± 20.37 ^b^	126.99 ^a^	0.0020
Mean duration of E2 (min)	24.55 ± 8.84 ^b^	11.80 ± 5.09 ^b^	25.39 ± 10.70 ^b^	52.48 ± 17.20 ^a^	0.0061
Time to first sustained E2 (h)	4.27 ± 0.41 ^ab^	5.21 ± 0.28 ^a^	4.81 ± 0.60 ^ab^	3.45 ± 0.42 ^b^	0.0474

The values for the numbers of Np, C, Pd, F, E1, E2 and G were square root transformed. The values for the duration waveform parameters of Np, C, Pd, F, G, E1, and time to first E, time to first E2, and time to first sustained E2 were done logarithmic transformation. Means within rows followed by different letters are significantly different (*p* < 0.05, Tukey’s test). “ns” indicates no significant difference. The values are presented as mean ± SE.

**Table 4:** Linear correlations between EPG parameters and winged *S. miscanthi* settled on wheat accessions and varieties 12, 24, 48, and 72 h after being released.

EPG Parameters	Percentage of Winged Aphids Settled on Wheat Accessions or Varieties after Different Infestation Times			
	12 h	24 h	48 h	72 h
Total duration of Np (min)	−0.31 *	−0.62 ***	−0.29 *	−0.34 *
Total duration of C (h)	−0.13	−0.17	−0.17	−0.17
Total duration of Pd (min)	−0.06	−0.08	−0.07	−0.09
Mean duration of E1(min)	−0.27 *	−0.31 *	−0.28 *	−0.27 *
Total duration of E2 (min)	0.40 *	0.53 ***	0.48 ***	0.50 ***
Total number of G (min)	0.14	0.12	0.17	0.07
Total duration of Np (min)	−0.31 *	−0.62 ***	−0.29 *	−0.34 *

Asterisks indicate significant difference (*, *p* < 0.05, ***, *p* < 0.001).

**Table 5:** Linear correlations between leaf trichome density and percentage of winged *S. miscanthi* settled on wheat accessions and varieties 12, 24, 48 and 72 h after being released.

Trichome Density (mm^2^)	Percentage of Winged Aphids Settled on Wheat Accessions after Different Infestation Times			
	12 h	24 h	48 h	72 h
Adaxial trichomes	−0.53 ***	−0.58 ***	−0.56 ***	−0.58 ***
Abaxial trichomes	−0.44 **	−045 **	−0.48 **	−0.47 **

Asterisks indicate significant difference (**, *p* < 0.01, ***, *p* < 0.001).

## Data Availability

Data can be provided upon request from the lead author.

## Supplementary Materials

The following are available online at https://www.mdpi.com/article/10.3390/plants11081094/s1, Table S1: Number of winged aphids settled per plant counted after 24 and 48 h after release to wheat seedlings, aphid resistance index, and aphid resistance scale of *S. miscanthi* on the wheat accessions and varieties screened for antixenosis resistance. Figure S1: Dendrogram clustering of 133 wheat accessions based on the number of winged *Sitobion miscanthi* settled 48 h after aphid release. Figure S2: A scheme of the rectangular gauge cage (2 × 2 × 1.5 m), wheat seedling, and winged *S. miscanthi* arrangement for the preliminary antixenosis resistance screening and aphid bioassay for further antixenosis resistance tests (choice test).
