# Supplementary material for: Screening and Evaluation for Antixenosis Resistance in Wheat Accessions and Varieties to Grain Aphid, Sitobion miscanthi (Takahashi) (Hemiptera: Aphididae)
Source: Plants (Basel). 2022 Apr 18;11(8):1094. doi: 10.3390/plants11081094 (PMC9031254; doi:10.3390/plants11081094)

Table S1. Plant species, origin, number of winged aphids settled per plant counted after 24 and 48 hours of release to wheat seedlings, aphid resistance index and aphid resistance scale of *Sitobion miscanthi* on the wheat accessions screened for antixenosis resistance.

Origin	Province	Wheat accessions	Number of winged *S. miscanthi* settled after 24 hours				Number of winged *S. miscanthi* settled after 48 hours			
			Number of aphids/ accessions	Resistance index	Resistance descriptions	Rank	Number of aphids/ accessions	Resistance index	Resistance descriptions	Rank
China	Beijing	Lunuan266	2.50	0.29	HR	1	2.00	0.29	HR	1
Ethiopia	Amhara	243726	2.50	0.29	HR	1	2.50	0.36	MR	2
Ethiopia	Tigray	213312	2.50	0.29	HR	1	2.50	0.36	MR	2
Ethiopia	Tigray	8324	3.00	0.35	MR	2	2.50	0.36	MR	2
Ethiopia	Tigray	243710	3.00	0.35	MR	2	2.50	0.36	MR	2
Ethiopia	Amhara	7407	3.00	0.35	MR	2	2.50	0.36	MR	2
Ethiopia	Amhara	7276	3.00	0.35	MR	2	3.00	0.43	MR	2
Ethiopia	Amhara	203971	3.00	0.35	MR	2	3.00	0.43	MR	2
Ethiopia	Tigray	207845	4.00	0.46	MR	2	3.00	0.43	MR	2
Ethiopia	Tigray	243714	3.50	0.40	MR	2	3.50	0.50	MR	2
Ethiopia	Amhara	7248	3.00	0.35	MR	2	3.50	0.50	MR	2
Ethiopia	Amhara	227068	4.00	0.46	MR	2	3.50	0.50	MR	2
Ethiopia	Amhara	216536	4.50	0.52	MR	2	3.50	0.50	MR	2
Ethiopia	Amhara	7298	4.00	0.46	MR	2	3.50	0.50	MR	2
Ethiopia	Amhara	222493	7.00	0.81	LR	3	3.50	0.50	MR	2
Ethiopia	Tigray	Mekelle -04	6.00	0.69	LR	3	3.50	0.50	MR	2
China	Beijing	Longmaizi-1	4.50	0.52	MR	2	3.50	0.50	MR	2
Ethiopia	Tigray	226251	4.50	0.52	MR	2	4.00	0.57	MR	2
Ethiopia	Amhara	203773	5.00	0.58	MR	2	4.00	0.57	MR	2
China	Beijing	Lunxuan145	5.50	0.64	LR	3	4.00	0.57	MR	2
China	Beijing	k28	8.00	0.92	LS	4	4.00	0.57	MR	2
China	Beijing	kechun-120832	4.50	0.52	MR	2	4.00	0.57	MR	2
Ethiopia	Amhara	5173	5.50	0.64	LR	3	4.00	0.57	MR	2
Ethiopia	Amhara	6844	7.50	0.87	LR	3	4.00	0.57	MR	2
Ethiopia	Amhara	243732	5.50	0.64	LR	3	4.00	0.57	MR	2
Ethiopia	Amhara	22625	3.00	0.35	MR	2	4.00	0.57	MR	2
Ethiopia	Amhara	206530	7.00	0.81	LR	3	4.50	0.65	LR	3
Ethiopia	Amhara	7281	5.00	0.58	MR	2	4.50	0.65	LR	3
Ethiopia	Tigray	Dandaa	8.50	0.98	LS	4	4.50	0.65	LR	3
Ethiopia	Tigray	207855	5.50	0.64	LR	3	4.50	0.65	LR	3
Ethiopia	Tigray	kingbird	6.00	0.69	LR	3	4.50	0.65	LR	3
China	Beijing	Kechun131031	7.00	0.81	LR	3	4.50	0.65	LR	3
Ethiopia	Amhara	204511	6.00	0.69	LR	3	4.50	0.65	LR	3
Ethiopia	Tigray	Daka	8.50	0.98	LS	4	4.50	0.65	LR	3
Ethiopia	Tigray	236293	8.50	0.98	LS	4	4.50	0.65	LR	3
Ethiopia	Amhara	227067	6.00	0.69	LR	3	4.50	0.65	LR	3
Ethiopia	Amhara	203968	8.50	0.98	LS	4	4.50	0.65	LR	3
Ethiopia	Amhara	206561	5.50	0.64	LR	3	4.50	0.65	LR	3
Ethiopia	Amhara	204363	7.50	0.87	LR	3	5.00	0.72	LR	3

Ethiopia	Amhara	7646	7.00	0.81	LR	3	5.00	0.72	LR	3
Ethiopia	Amhara	203842	6.00	0.69	LR	3	5.00	0.72	LR	3
Ethiopia	Amhara	226256	3.00	0.35	MR	2	5.00	0.72	LR	3
Ethiopia	Amhara	kakaba	4.00	0.46	MR	2	5.00	0.72	LR	3
China	Beijing	Lunxuan166	8.50	0.98	LS	4	5.00	0.72	LR	3
China	Beijing	Lunxuan103	7.50	0.87	LR	3	5.00	0.72	LR	3
China	Beijing	Kechun110182	10.50	1.21	MS	5	5.00	0.72	LR	3
Ethiopia	Tigray	243720	12.00	1.39	MS	5	5.00	0.72	LR	3
Ethiopia	Tigray	Mekelle-01	6.00	0.69	LR	3	5.00	0.72	LR	3
Ethiopia	Tigray	204409	8.00	0.92	LS	4	5.00	0.72	LR	3
Ethiopia	Amhara	8231	8.00	0.92	LS	4	5.00	0.72	LR	3
China	Beijing	Kechun13290	11.00	1.27	MS	5	5.50	0.79	LR	3
China	Beijing	Lunxuan45	5.00	0.58	MR	2	5.50	0.79	LR	3
China	Beijing	Lunxuan061	7.00	0.81	LS	4	5.50	0.79	LR	3
Ethiopia	Tigray	Wane	3.50	0.40	MR	2	5.50	0.79	LR	3
China	Beijing	Yong 36021	8.00	0.92	LS	4	5.50	0.79	LR	3
Ethiopia	Amhara	5171	8.00	0.92	LS	4	5.50	0.79	LR	3
Ethiopia	Tigray	221740	18.00	2.08	HS	6	5.50	0.79	LR	3
Ethiopia	Tigray	216631	6.00	0.69	LR	3	5.50	0.79	LR	3
Ethiopia	Tigray	222439	5.00	0.58	MR	2	5.50	0.79	LR	3
Ethiopia	Amhara	216537	7.50	0.87	LR	3	5.50	0.79	LR	3
Ethiopia	Tigray	Tay	4.00	0.46	MR	2	5.50	0.79	LR	3
Ethiopia	Tigray	213320	5.00	0.58	MR	2	6.00	0.86	LR	3
China	Beijing	W23	3.50	0.40	MR	2	6.00	0.86	LR	3
Ethiopia	Tigray	216448	3.00	0.35	MR	2	6.00	0.86	LR	3
Ethiopia	Amhara	7565	5.00	0.58	MR	2	6.50	0.93	LS	4
Ethiopia	Amhara	Lunxuan16	4.00	0.46	MR	2	6.50	0.93	LS	4
Ethiopia	Amhara	204476	8.50	0.98	LS	4	6.50	0.93	LS	4
Ethiopia	Tigray	222821	7.50	0.87	LR	3	6.50	0.93	LS	4
Ethiopia	Amhara	206564	11.50	1.33	MS	5	6.50	0.93	LS	4
Ethiopia	Amhara	206604	8.50	0.98	LS	4	6.50	0.93	LS	4
Ethiopia	Amhara	206590	8.00	0.92	LS	4	6.50	0.93	LS	4
Ethiopia	Amhara	226944	5.50	0.64	LR	3	6.50	0.93	LS	4
Ethiopia	Amhara	7285	9.00	1.04	LS	4	7.00	1.01	LR	4
Ethiopia	Amhara	243697	5.00	0.58	MR	2	6.50	0.93	LS	4
China	Beijing	Lunxuan66	9.50	1.10	LS	4	6.50	0.93	LS	4
Ethiopia	Tigray	Alidero	10.00	1.16	LS	4	6.50	0.93	LS	4
Ethiopia	Tigray	Mekelle-03	9.00	1.04	LR	3	7.00	1.01	LS	4
China	Beijing	2018NiaNpin	8.50	0.98	LS	4	7.00	1.01	LS	4
Ethiopia	Amhara	227055	9.50	1.10	LS	4	7.00	1.01	LS	4
Ethiopia	Tigray	243723	4.00	0.46	MR	2	7.00	1.01	LS	4
Ethiopia	Amhara	7279	4.00	0.46	MR	2	7.00	1.01	LS	4
Ethiopia	Amhara	216544	12.00	1.39	MS	2	7.00	1.01	LS	4
Ethiopia	Tigray	206533	11.00	1.27	MS	2	7.00	1.01	LS	4
Ethiopia	Tigray	7946	8.50	0.98	LS	4	7.00	1.01	LS	4
Ethiopia	Amhara	206620	9.50	1.10	LS	4	7.00	1.01	LS	4
Ethiopia	Amhara	216542	7.00	0.81	LR	3	7.00	1.01	LS	4
Ethiopia	Tigray	216543	12.00	1.39	MS	5	7.50	1.08	LS	4
Ethiopia	Amhara	Ogolcho	11.00	1.27	MS	5	7.50	1.08	LS	4
Ethiopia	Amhara	243704	10.50	1.21	MS	5	7.50	1.08	LS	4
Ethiopia	Amhara	222494	10.00	1.16	LS	4	7.50	1.08	LS	4
Ethiopia	Amhara	203755	11.00	1.27	MS	5	8.00	1.15	LS	4

China	Beijing	Lunxuan6	9.00	1.04	LS	4	8.00	1.15	LS	4
Ethiopia	Tigray	243721	9.50	1.10	LS	4	8.00	1.15	LS	4
Ethiopia	Amhara	7451	12.00	1.39	MS	5	8.50	1.22	MS	5
Ethiopia	Amhara	5215	17.50	2.02	HS	6	8.50	1.22	MS	5
Ethiopia	Amhara	243696	10.50	1.21	MS	5	8.50	1.22	MS	5
China	Beijing	Lunxuan13	15.50	1.79	HS	6	8.50	1.22	MS	5
Ethiopia	Amhara	204585	13.00	1.50	MS	5	8.50	1.22	MS	5
Ethiopia	Tigray	203703	11.50	1.33	MS	5	8.50	1.22	MS	5
Ethiopia	Amhara	203754	10.50	1.21	MS	5	9.00	1.29	MS	5
Ethiopia	Tigray	Hidase	7.00	0.81	LR	4	9.00	1.29	MS	5
Ethiopia	Amhara	7938	13.00	1.50	MS	5	9.00	1.29	MS	5
Ethiopia	Tigray	213302	8.00	0.92	LS	4	9.00	1.29	MS	5
Ethiopia	Amhara	204014	10.00	1.16	LS	4	9.00	1.29	MS	5
Ethiopia	Tigray	216540	9.50	1.10	LS	4	9.50	1.36	MS	5
Ethiopia	Amhara	206593	12.00	1.39	MS	5	9.50	1.36	MS	5
Ethiopia	Tigray	243711	11.00	1.27	MS	5	10.00	1.44	MS	5
Ethiopia	Amhara	8255	12.00	1.39	MS	5	10.00	1.44	MS	5
Ethiopia	Amhara	7650	9.50	1.10	LS	4	10.00	1.44	MS	5
Ethiopia	Tigray	Paven76	14.50	1.68	HS	6	10.00	1.44	MS	5
Ethiopia	Amhara	243734	5.00	0.58	MR	5	10.00	1.44	MS	5
Ethiopia	Tigray	7253	16.00	1.85	HS	6	10.00	1.44	MS	5
China	Beijing	Lunxuan146	11.00	1.27	MS	5	10.50	1.51	HS	6
Ethiopia	Amhara	216553	10.00	1.16	LS	4	11.00	1.58	HS	6
Ethiopia	Tigray	206535	10.50	1.21	MS	5	11.50	1.65	HS	6
Ethiopia	Amhara	7259	25.00	2.89	HS	6	11.50	1.65	HS	6
Ethiopia	Tigray	213290	16.00	1.85	HS	6	11.50	1.65	HS	6
Ethiopia	Amhara	243702	10.00	1.16	LS	4	11.50	1.65	HS	6
Ethiopia	Tigray	243722	18.00	2.08	HS	6	12.00	1.72	HS	6
Ethiopia	Amhara	7257	19.50	2.25	HS	6	12.00	1.72	HS	6
Ethiopia	Amhara	216533	11.50	1.33	MS	5	12.00	1.72	HS	6
Ethiopia	Amhara	222292	13.50	1.56	HS	6	12.00	1.72	HS	6
China	Beijing	Kechun150859	7.00	0.81	LR	4	12.50	1.80	HS	6
Ethiopia	Amhara	243727	14.50	1.68	HS	6	12.50	1.80	HS	6
Ethiopia	Amhara	7122	10.50	1.21	MS	5	12.50	1.80	HS	6
China	Beijing	Beijing 837	13.00	1.50	MS	5	12.50	1.80	HS	6
Ethiopia	Amhara	206598	18.00	2.08	HS	6	13.50	1.94	HS	6
Ethiopia	Tigray	236292	13.00	1.50	MS	5	14.50	2.08	HS	6
Ethiopia	Tigray	Shehan	12.50	1.45	MS	5	16.00	2.30	HS	6
Ethiopia	Amhara	243731	19.00	2.20	HS	6	16.00	2.30	HS	6
Ethiopia	Amhara	231487	23.00	2.66	HS	6	16.50	2.37	HS	6
Ethiopia	Tigray	Dereselign	11.00	1.27	MS	5	17.00	2.44	HS	6
Ethiopia	Amhara	204586	13.00	1.50	MS	5	25.00	3.59	HS	6

N.B: Where, HR=highly resistance, MR= moderately resistance, LR= Lowly resistance, LS= Lowly susceptible, MS= moderately susceptible and HS= highly susceptible

Figure S1. Dendrogram clustering of 133 wheat accessions based on the number of winged *Sitobion miscanthi* settled after 48 hours of aphid released.

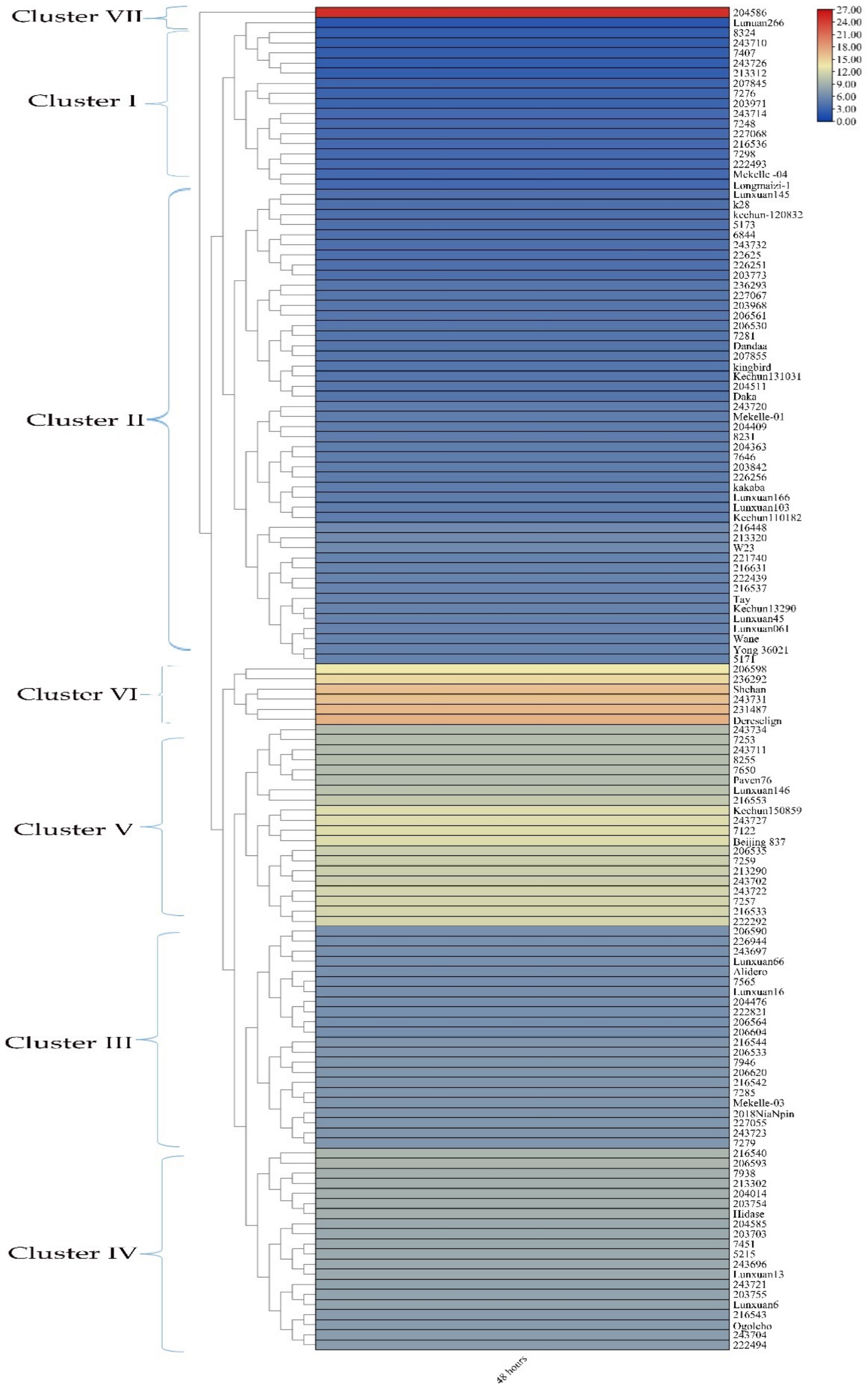

Figure S2. Seedling arrangement for preliminary antixenosis resistance Screening and aphid bioassay for further antixenosis test (choice test) inside m x 2 m x 1.5 m rectangular gauze cage. Note: doors were covered with black plastic.

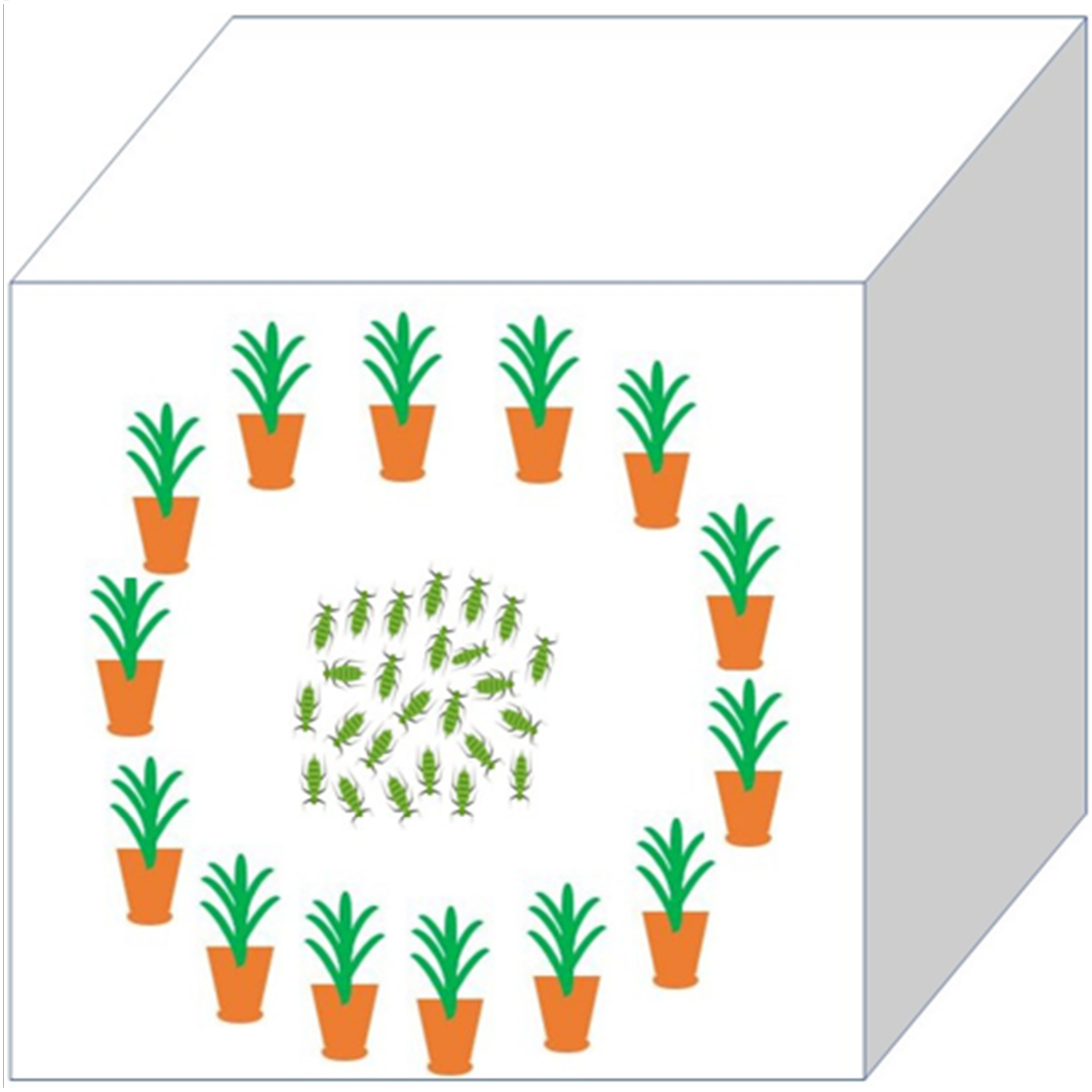

Supplement: Supplementary file 1 [file plants-11-01094-s001.zip › plants-1678147-supplementary.pdf]
